# Assessing the Effectiveness of the Respecting the Circle of Life Project on Condom and Contraception Self-efficacy Among American Indian Youth

**DOI:** 10.1007/s11121-023-01514-4

**Published:** 2023-05-25

**Authors:** Jaime L. Begay, Rachel A. Chambers, Summer Rosenstock, Christopher G. Kemp, Angelita Lee, Francene Lazelere, Laura Pinal, Lauren Tingey

**Affiliations:** 1 Johns Hopkins Center for Indigenous Health, Johns Hopkins Bloomberg School of Public Health, 327 Loloma Street, Tuba City, AZ 86045 USA; 2 Johns Hopkins Center for Indigenous Health, Johns Hopkins Bloomberg School of Public Health, 415 North Washington Street, Baltimore, MD 21231 USA; 3 Johns Hopkins Center for Indigenous Health, Johns Hopkins Bloomberg School of Public Health, 102 General Crook Street, Fort Apache, AZ 85926 USA

**Keywords:** American Indian, Youth, RCT, Prevention, Condom, Contraception, Self-efficacy

## Abstract

Respecting the Circle of Life (RCL) is a teen pregnancy prevention program that was evaluated for effectiveness on sexual health risk behaviors through a two-arm randomized control trial (RCT) with American Indian (AI) youth ages 11–19. The objective of this study is to investigate the effects of RCL compared to a control group on items of condom and contraception self-efficacy. Linear regression analysis was used to compare differences in each item that included condom and contraception self-efficacy scales among the intervention and control participants at baseline, 3 and 9 months post intervention. Youth enrolled in the intervention reported higher levels of condom and contraception self-efficacy across almost all individual items. Exceptions include items related to partner negotiation of condom self-efficacy at 3 months (*p* = 0.227) and 9 months (*p* = 0.074) post intervention. Findings indicate RCL is effective at improving overall condom and contraception self-efficacy but did not impact the specific component of partner negotiation for either condom or contraception self-efficacy. This inquiry provides rationale to further explore components of RCL related to partner negotiation.

## Introduction

American Indian (AI) adolescents contend with one of the highest rates of teen pregnancy of all race and ethnicities in the US (Indian Health Service, 2014). In 2020, the birth rate for American Indian/Alaskan Native (AI/AN) teens (age 15–19) was 25.7 per 1000 women, well above the birth rate of whites (10.4) and all races (15.4) (Osterman et al., 2022). Many AI females (41%) begin childbearing in adolescence and compared to the general US population, bear twice as many children as teens (Eaton et al., 2012). Compared with all US races, AI adolescents were more likely to have had sex for the first time before age 13 (all US races, 6% vs AI, 11%) and are more likely to have ever had sex (all US races, 47% vs AI, 69%) (Centers for Disease Control & Prevention, 2012; Indian Health Service, 2001, 2009). Furthermore, sexually transmitted infection (STI) rates are high among AI/ANs; chlamydia and gonorrhea rates among AIs were 3.7 and 4.6 times higher than that of whites in 2018 (Centers for Disease Control & Prevention, 2018). AI youth have greater reproductive health disparities; therefore, interventions that reduce risk for teen pregnancy and STIs are imperative.

Several evidence-based interventions (EBIs) in the US have shown to be efficacious in STI and pregnancy prevention (DiClemente et al., 2004; Rotheram-Borus et al., 2003; St Lawrence et al., 1995). A study focused on African American adolescents posits that a culturally and gender tailored intervention may enhance skills and preventative behaviors that reduce pregnancy and STIs (DiClemente et al., 2004). Additionally, interventions rooted in theoretical frameworks promote the adoption of protective health behaviors, such as condom use and safe sex (DiClemente et al., 2008; Rotheram-Borus et al., 2003). On a broader spectrum, some programs have had small effect sizes and failed to replicate, notably within populations outside of the original studies that demonstrated evidence (Juras et al., 2019). Unintended pregnancy in AI youth remains a public health concern, and little is known about rigorously tested sexual health interventions that cater to AI youth (Centers for Disease Control & Prevention, 2021). To address this research gap, the continued adaptation and assessment of EBIs that are designed with and for AI youth are imperative.

Respecting the Circle of Life (RCL) program was designed with and for Native youth ages 11–19. A rigorous randomized controlled trial (RCT) of the RCL intervention was conducted with youth from a tribal community in Arizona. This evaluation found that participants randomized to the intervention had significantly better condom (*p* = 0.024) and contraceptive self-efficacy (*p* < 0.001) than the control at 9 months (Tingey et al., 2021). While RCL’s impact on overall condom and contraception self-efficacy is encouraging, predictors are multifaceted consisting of items including access to, correct usage, ability to seek help to obtain and partner negotiation regarding condoms and contraception. There has been little inquiry into the impact of RCL on the items of condom use predictors.

Studies indicate youth need to be proficient in all individual items of condom and contraception self-efficacy to implement the actual behavior of condom and/or contraception (Longmore et al., 2003). Thus, there are multiple pieces that correspond with the individual items that culminate the overarching constructs (condom and contraception self-efficacy) of interest. First, youth need to know where and how to access condoms and contraception (Amialchuk & Gerhardinger, 2015). Condom accessibility remains a critical component to actual condom and safer sexual behavior (Widman et al., 2014). Likewise, when youth are knowledgeable about contraceptives including condoms and their benefits, they have the confidence in their ability to obtain and use condoms (Ritchwood et al., 2017). In addition to accessing contraception methods, communication with a parent or trusted adult (TA) has been associated with actual contraception use indicating the ability to ask a TA about condoms and contraception (Amialchuk & Gerhardinger, 2015). Finally, the ability to talk with a partner about condoms and sexual health are predictive of actual condom and contraception use (Tschann et al., 2010; Amialchuk & Gerhardinger, 2015). Studies indicate these conversations are difficult and often embarrassing for youth who have minimal intimate sexual experiences but remain important (Widman et al., 2014). In a study focused on Latino youth, partner communication and negotiation about condoms increased the likelihood of condom use (Tschann et al., 2010). Furthermore, in a study with youth in grades 7–12, those who discussed contraception with their partners before sex were more than twice as likely to use contraception than those who did not (Manlove et al., 2003). Programs to increase condom and contraception self-efficacy, including RCL, should focus on all individual items of condom and contraceptive self-efficacy as they are all important in promotion of actual condom and contraception self-efficacy.

The overall objective of this study was to establish the efficacy of the RCL program in improving the various items of condom and contraception self-efficacy. We hypothesize that each item of condom and contraception self-efficacy will be significantly higher for RCL intervention participants compared to the control participants at 9 months post intervention. Results will provide deeper understanding of the overarching impact of RCL on these important predictors of condom and contraception self-efficacy.

## Methods

This project includes the evaluation of RCL through a randomized control trial design and specifically assesses the impact of the RCL program on condom and contraception self-efficacy items. Our goal is to answer the research question: Which individual items of condom and contraception self-efficacy are significantly different among youth assigned to the RCL intervention compared to AI youth assigned to a control group at 3 and 9 months post program completion. The study design was approved by the participating tribal community’s governing Tribal Council, Health Board, and Johns Hopkins Institutional Review Board. This manuscript was approved by the participating community’s Health Board and Tribal Council.

### Participants

Youth were recruited through public service announcements, school advertising, and public outreach events utilizing non-probability sampling (Tingey et al., 2015). Inclusion criteria were (1) between 11 and 19 years old; (2) living on or near the participating tribal nation; and (3) ability to participate in the entire intervention. The exclusion criteria included (1) inability to participate in full intervention and (2) unwilling to be randomized. To enroll, youth ≥ 18 years old required a signed informed consent and for youth < 18 years old, parental permission and youth assent were required (Tingey et al., 2015). After completing informed consent, parental permission, and assent, the youth selected a trusted adult (many but not all of whom were parents) to enroll with them in the study. The trusted adult completed informed consent to participate.

Once enrolled, study participants (youth/trusted adult dyad) were randomized 1:1 to the intervention or control group. Randomization was stratified by age group (11–12, 13–15, or 16–19 years) and sex and carried out at the individual level, using block randomization. Participants were blinded to randomization status. Once randomized, participants formed self-selected same-sex peer-groups of 8 to 10 participants each.

### Study Setting

The study setting was a basketball summer camp offered at different local schools within an Arizona tribal nation. Youth signed up for the basketball camp and, if consent and assent were granted, they were randomly assigned the youth condition, which determined the location of the basketball camp they attended. The schools were not close to one another, which helped prevent intervention delivery contamination (Tingey et al., 2017).

The RCL intervention is a risk reduction intervention for AI youth and families. RCL is a 9-session curriculum that promotes discussion of condom use, abstinence, and sexually transmitted diseases (STD) and HIV prevention. The first 8 sessions are each ~2 h in length and delivered to small groups of same sex self-selected peer groups. The 9th session is taught to youth and a trusted adult through a home visit. The comparison (control) condition includes Healthy Youth (HY), a 9-lesson program delivered to AI youth ages 11–19 and families in the same structure format as RCL. The control group received 9 educational lessons on nutrition, fitness, outdoor recreation, safety, and environmental protection. Youth received the control program in the same format as the RCL intervention (Tingey et al., 2017).

### Data Collection

The baseline assessment was conducted after informed consent and immediately before randomization via self-report. Data were collected either via tablet/computer or paper with data entry by study staff. Follow-up assessments were collected by independent evaluators at 3 and 9 months post intervention in a private location of the participant’s choosing.

Youth participants completed the Youth Health Risk Behavioral Inventory (YHRBI) (Tingey et al., 2017). The YHRBI documented demographic information and knowledge, intentions, prior experience with regard to protective and risk behaviors including the focus of this analysis: condom and contraception self-efficacy. The inventory was administered at baseline, 3 and 9 months post intervention. All assessments were self-report, and data was analyzed through the STATA program (StataCorp, 2021).

The primary outcomes of interest include six condom and six contraception self-efficacy items. The variables for condom self-efficacy included the following: (1) Get condoms, (2) Put a condom on correctly, (3) Convince partner to use condoms, (4) Ask for condoms in store, (5) Ask for condoms at Indian Health Service (IHS), and (6) Refuse sex if partner will not use condom (Table 1). The variables for contraception self-efficacy included the following: (1) Get myself or partner birth control protection not including condoms, (2) Use birth control correctly other than condoms, (3) Could convince partner to use birth control even if they do not want to, (4) Could ask parent/trusted adult for help getting birth control, (5) Could ask for birth control at IHS, and (6) Could refuse sex if partner will not use birth control (Table 2) (Tingey et al., 2017). In this assessment, contraception did not include condoms; instead in the assessment, examples were provided of what contraception meant (e.g., Patch, Pills, Ring). Response options for each item were based on a Likert scale 1–5; where 1 = Yes, I could, 2 = Maybe I could, 3 = Don’t know, 4 = Probably, I could not, and 5 = No, I could not (Table 3) (Tingey et al., 2017). Items were reverse coded prior to analysis.

**Table 1:** Condom use self-efficacy among American Indian Youth in the Respecting the Circle of Life project (*N* = 534)

Items	Group size N interven; N control	Missing %	Intervention mean (SEM)	Control mean (SEM)	AMD (95% CI)	*p* value	AMD, Int*Sex (95% CI)	*p* value
	**Get condoms**							
Baseline	248;253	6.2%	2.96 (0.08)	2.94 (0.08)	0.02 (−0.21–0.25)	0.8677	0.16 (−0.29–0.62)	0.5
3 months	219;222	4.8%	4.04 (0.08)	3.50 (0.08)	0.54 (0.32–0.77)	< 0.0001	−0.13 (−0.59–0.32)	0.6
9 months	209;222	4.2%	4.14 (0.08)	3.53 (0.08)	0.61 (0.39–0.83)	< 0.0001	−0.02 (−0.46–0.42)	0.9
	**Put on condoms**							
Baseline	244;244	8.6%	2.95 (0.08)	2.88 (0.08)	0.07 (−0.15–0.28)	0.5401	0.14 (−0.30–0.57)	0.5
3 months	220;219	5.2%	4.05 (0.08)	3.21 (0.08)	0.84 (0.62–1.05)	< 0.0001	0.13 (−0.31–0.56)	0.6
9 months	209;220	4.7%	4.10 (0.08)	3.24 (0.08)	0.86 (0.64–1.07)	< 0.0001	0.10 (−0.33–0.53)	0.6
	**Convince partner use condoms**							
Baseline	245;247	7.9%	3.40 (0.08)	3.39 (0.08)	0.01 (−0.20–0.23)	0.9007	0.24 (−0.18–0.67)	0.3
3 months	223;223	3.7%	4.16 (0.07)	4.04 (0.07)	0.13 (−0.07–0.32)	0.2128	−0.33 (−0.72–0.06)	0.10
9 months	211;221	4.0%	4.20 (0.07)	3.92 (0.07)	0.28 (0.09–0.47)	0.0033	−0.14 (−0.52–0.23)	0.5
	**Ask condom store**							
Baseline	243;251	7.5%	2.83 (0.08)	2.75 (0.08)	0.08 (−0.14–0.31)	0.4524	0.12 (−0.32–0.57)	0.6
3 months	223;224	3.5%	3.65 (0.09)	2.98 (0.09)	0.67 (0.43–0.91)	< 0.0001	0.13 (−0.35–0.61)	0.6
9 months	211;223	3.6%	3.63 (0.08)	3.10 (0.08)	0.53 (0.30–0.76)	< 0.0001	0.07 (−0.39–0.54)	0.8
	**Ask condom IHS**							
Baseline	242;252	7.5%	2.99 (0.08)	3.01 (0.08)	−0.02 (−0.25–0.21)	0.8641	0.28 (−0.17–0.74)	0.2
3 months	223;224	3.5%	4.06 (0.08)	3.44 (0.08)	0.62 (0.40–0.84)	< 0.0001	−0.07 (−0.52–0.37)	0.7
9 months	211;222	3.8%	4.10 (0.08)	3.36 (0.08)	0.73 (0.51–0.95)	< 0.0001	0.09 (−0.35–0.53)	0.7
	**Refuse sex if partner will not use condom**							
Baseline	243;244	8.8%	3.48 (0.09)	3.63 (0.09)	−0.14 (−0.39–0.10)	0.2497	0.10 (−0.39–0.59)	0.7
3 months	221;222	4.3%	4.28 (0.08)	4.15 (0.08)	0.13 (−0.08–0.35)	0.2273	0.02 (−0.41–0.46)	0.9
9 months	211;224	3.3%	4.27 (0.08)	4.07 (0.08)	0.19 (−0.02–0.41)	0.0741	−0.06 (−0.49–0.37)	0.8

**Table 2:** Contraception use self-efficacy among American Indian Youth in the Respecting the Circle of Life project (*N* = 534)

Items	Group size N interven; N control	Missing %	Intervention mean (SEM)	Control mean (SEM)	AMD (95% CI)	*p* value	AMD, Int*Sex (95% CI)	*p* value
	**Get Contraception not condom**							
Baseline	242;241	9.6%	3.05 (0.08)	3.16 (0.08)	−0.11 (−0.33–0.11)	0.3160	0.06 (−0.38–0.50)	0.8
3 months	222;223	3.9%	3.73 (0.08)	3.36 (0.08)	0.37 (0.15–0.59)	0.0012	−0.20 (−0.64–0.24)	0.4
9 months	211;223	3.6%	3.64 (0.07)	3.42 (0.07)	0.22 (0.02–0.43)	0.0338	−0.12 (−0.53–0.29)	0.6
	**Use contraception correct other than condoms**							
Baseline	242;242	9.4%	2.98 (0.08)	3.07 (0.08)	−0.10 (−0.31–0.11)	0.3653	0.10 (−0.32–0.52)	0.6
3 months	221;220	4.8%	3.72 (0.08)	3.35 (0.08)	0.37 (0.15–0.59)	0.0009	0.18 (−0.26–0.61)	0.4
9 months	209;222	4.2%	3.66 (0.07)	3.28 (0.07)	0.39 (0.18–0.59)	0.0002	0.15 (−0.26–0.56)	0.5
	**Could convince partner use birth control even if they don’t want to**							
Baseline	242;245	8.8%	3.15 (0.08)	3.21 (0.08)	−0.06 (−0.27–0.15)	0.5831	0.06 (−0.37–0.49)	0.8
3 months	222;221	4.3%	3.93 (0.07)	3.71 (0.07)	0.22 (0.01–0.42)	0.0383	−0.29 (−0.70–0.12)	0.2
9 months	210;225	3.3%	3.89 (0.07)	3.58 (0.07)	0.31 (0.12–0.51)	0.0019	0.26 (−0.13–0.65)	0.2
	**Ask parent/TA for help getting contraception**							
Baseline	243;242	9.2%	3.04 (0.08)	3.08 (0.08)	−0.04 (−0.26–0.19)	0.7335	0.03 (−0.42–0.48)	0.9
3 months	223;221	4.1%	3.78 (0.08)	3.48 (0.08)	0.30 (0.08–0.53)	0.0086	0.20 (−0.25–0.65)	0.4
9 months	208;224	4.0%	3.65 (0.08)	3.31 (0.08)	0.34 (0.12–0.56)	0.0028	0.22 (−0.23–0.66)	0.3
	**Ask contraception IHS**							
Baseline	244;246	8.2%	3.05 (0.08)	3.09 (0.08)	−0.04 (−0.26–0.18)	0.7134	0.02 (−0.43–0.47)	0.9
3 months	223;219	4.5%	3.90 (0.08)	3.50 (0.08)	0.41 (0.19–0.62)	0.0002	−0.01 (−0.44–0.41)	0.9
9 months	208;222	4.4%	3.77 (0.08)	3.30 (0.08)	0.47 (0.25–0.70)	< 0.0001	0.15 (−0.31–0.60)	0.5
	**Refuse sex if partner no contraception**							
Baseline	236;237	11.4%	3.35 (0.09)	3.54 (0.08)	−0.19 (−0.43–0.05)	0.1152	−0.01 (−0.49–0.46)	1
3 months	222;221	4.3%	4.21 (0.08)	3.94 (0.08)	0.27 (0.05–0.49)	0.0163	0.24 (−0.20–0.67)	0.3
9 months	208;225	3.8%	4.23 (0.08)	3.88 (0.07)	0.35 (0.14–0.56)	0.0013	0.09 (−0.33–0.51)	0.7

**Table 3:** Items with accompanying scoring Likert scale

		**Scoring**
**Condom self-efficacy questions within subscale**		
D1	I could get condoms	
D2	I could put a condom on correctly	
D3	I could convince my partner that we should use a condom even if he or she doesn’t want to	
D4	I could ask for condoms in a store	
D5	I could ask for condoms at IHS	Range 1–5:
D6	I could refuse to have sex if my partner will not use a condom	
**Contraception self-efficacy questions within subscale**		1 = Yes, I could 2 = Maybe, I could 3 = Don’t know 4 = Probably, I could not 5 = No, I could not
D7	I could get myself or my partner a form of birth control other than condoms (e.g., pills, patch, ring, IUD, etc.)	
D8	Other than condoms, I could use birth control correctly (e.g., follow instructions)	
D9	I could convince my partner that we should use birth control even if they don’t want to	
D10	I could ask my partner or a trusted adult for help with getting birth control	
D11	I could ask for birth control at IHS	
D12	I could refuse to have sex if my partner will not use birth control	

### Statistical Analysis

Intent to treat analysis was utilized according to randomization assignment. A linear regression was conducted at different timepoints (baseline, 3 and 9 months) to assess the impact of RCL compared to control on outcomes of self-efficacy. All models controlled for baseline sex and age. Due to statistical significance (*p* < 0.05) differences in baseline values, we controlled for baseline responses to the item. Post hoc analyses tested for moderation of intervention effects by participant sex; estimates for an interaction between sex and trial arm are reported. Missing data was addressed by the following: (1) documentation; (2) treatment dropouts assessed for the intent to treat analysis; and (3) sensitivity analysis to compare missing data speculations (Tingey et al., 2017). The results indicate the higher the number, the higher the self-efficacy. The between group adjusted mean differences (AMD) with 95% confidence intervals was reported. We reported *p* values with a 0.05 threshold for statistical significance.

## Results

A total of 534 youth participants enrolled in the study (266 intervention; 268 control), among which 52.6% were girls and 47.4% were boys. The mean age was 13.27 (sd: 1.81) years at baseline, and all the participants self-reported race/ethnicity as American Indian. Youth participants were enrolled between May 2016 and June 2018. Participant descriptive statistics are reported in more detail in the manuscript reporting the trial primary outcomes (Tingey et al., 2021).

### Condom Self-efficacy (Table 1)

RCL participants reported higher condom self-efficacy than the control youth across most items at 3 and 9 months post. Youth receiving RCL had significantly higher scores than the control at 3 months, and these were sustained at 9 months for the following items: how to get condoms (mean 4.04 vs. 3.50 at 3 months, 4.14 vs. 3.53 at 9 months, *p* < 0.001 at both time points), how to put a condom on correctly (mean 4.05 vs. 3.21 at 3 months, 4.10 vs. 3.24 at 9 months, *p* < 0.001 at both time points), ask for condoms at store (mean 3.65 vs. 2.98 at 3 months, 3.63 vs. 3.10 at 9 months, *p* < 0.001 at both time points), and ask for condoms at Indian Health Services (mean 4.06 vs. 3.44 at 3 months, 4.10 vs. 3.36 at 9 months, *p* < 0.001 at both time points). There was no statistically significant difference for the item: refuse sex if partner will not use condom (mean 4.28 vs. 4.15 at 3 months, 4.27 vs. 4.07 at 9 months, *p* = 0.227 and *p* = 0.074, respectively) between the RCL and control groups. While another item: convince partner to use condoms, showed no statistical significance at 3 months (mean 4.16 vs. 4.04, *p* = 0.212); however at 9 months, there was a significant difference (mean 4.20 vs. 3.92, *p* = 0.003). No evidence of moderation by sex was identified.

### Contraception Self-efficacy (Table 2)

RCL participants reported higher self-efficacy across all contraception self-efficacy items at 3 and 9 months post. Youth receiving RCL had significantly higher scores than the control at 3 months, and these were sustained at 9 months for all items: getting contraception not condoms (mean 3.73 vs. 3.36 at 3 months, 3.64 vs. 3.42 at 9 months, *p* = 0.001 and *p* = 0.033, respectively), use contraception other than condoms correctly (mean 3.72 vs. 3.35 at 3 months, 3.66 vs. 3.28 at 9 months, *p* = 0.001 and *p* < 0.001, respectively), could convince partner to use birth control even if they do not want to (mean 3.93 vs. 3.71 at 3 months, 3.89 vs. 3.58 at 9 months, *p* = 0.038 and *p* = 0.002, respectively), ask parent/trusted adult for help in getting contraception (mean 3.78 vs. 3.48 at 3 months, 3.65 vs. 3.31 at 9 months, *p* = 0.009 and *p* = 0.003, respectively), ask for contraception at IHS (mean 3.90 vs. 3.50 at 3 months, 3.77 vs. 3.30 at 9 months, *p* < 0.001 at both time points), and refuse sex if partner has no contraception (mean 4.21 vs. 3.94 at 3 months, 4.23 vs. 3.88 at 9 months, *p* = 0.016 and *p* = 0.001, respectively). No evidence of moderation by sex was identified.

## Discussion

Results indicate the RCL program had statistically significant impacts on many of the individual items that make up condom and contraception self-efficacy at 3- and 9-month follow-up. All significant impacts at 3 months were sustained up to 9 months.

RCL shows promise in significantly improving youth’s reported ability to access both condoms and contraception generally and to ask for it at IHS. Tribal communities are close knit, often with close familial ties which may make it difficult for youth to ask for contraception or condoms due to confidentiality concerns (Fisher & Ball, 2003; Pampati et al., 2019). Thus, RCL reported higher self-efficacy pertaining to obtaining condoms and contraception at IHS than control youth is promising as it may indicate RCL helps youth to overcome previously reported concerns about seeking reproductive and sexual health care at IHS including the perceived lack of privacy within IHS (Tingey et al., 2019; Strom Chambers, 2021).

The difference in ability to obtain and ask for condoms and contraception at 3 months and 9 months post intervention may be due to the overall RCL curriculum or may be contributed to one or many of the following key components of RCL. (1) Activities that increase general knowledge about condoms and contraception including a review of types of birth control. Previous studies have shown increasing knowledge about available contraception is one important component to increasing uptake and adherence (Tomaszewski et al., 2017). Thus, combined with findings from Tingey et al., in which youth who received RCL reported higher knowledge at post intervention, we conclude that activities to improve knowledge about reproductive health may influence self-efficacy and eventually uptake of contraception and condom use. Future studies are needed to explore this association. (2) Activities that increase youth’s knowledge about where to obtain birth control and contraception including a discussion of where to get condoms and a list of resources/places with contact information about where condoms can be acquired. Again, studies have found increasing knowledge about where and how to get condoms and contraception may increase uptake and self-efficacy around obtaining these (Patterson et al., 2022). (3) Familiarity of IHS staff due to IHS employee presence at one of the lessons in which different types of birth control are presented. Recommendations to improve contraception uptake include establishing rapport with patients (Gavin et al., 2014). There is potential that having a provider or nurse introduce themselves and be available for questioning during an RCL session may be a first step to establishing rapport and becoming more comfortable talking about contraception with IHS employees. Since there are often limited places to obtain contraception in rural tribal communities, future studies should explore how each of these RCL activities increases confidence and ability to ask for contraception at IHS or other local clinics as this could inform future efforts to increase contraception uptake among AI youth.

It is also promising that RCL increased youth’s reported ability to ask their parent/trusted adult for help getting contraception. Parent/trusted adults are in a unique position to talk about sex with their child across their teenage years (Manning et al., 2009). Youth-parent communication can positively impact youth sexual behaviors, including contraceptive uptake and consistent condom use throughout adolescence (Akers et al., 2011; Ritchwood et al., 2017). At baseline in this study, levels of communication between youth and parent/trusted adult were low. When asked how often do you talk to your youth about how to use and how to get contraception, enrolled parent/trusted adults reported an average of 1.7 out of a 5 point Likert scale (1 = Never and 5 = Often) with less than one in five reporting they often or sometimes talked to their youth about either of these (Chambers et al., 2022). This lack of communication may be attributed to parent/trusted adult feeling they do not have the information or lack comfort in speaking with their youth about sexual health (Jaccard et al., 2000; Ashcraft & Murray, 2017). In a previous analysis, parent/trusted adults randomized to RCL reported more frequent conversations with their youth about contraception after completing RCL (see Chambers et al., 2022). Combined with our findings, these results suggest youth who receive RCL are better able to discuss contraception with their parent/trusted adult. Often, parent/trusted adults have trouble initiating conversations about sexual health and contraception with their youth (Raffaelli et al., 1998; Holtzman & Rubinson, 1995). RCL activities, specifically the parent/trusted adult lesson taught with the youth in which the parent/trusted adult discuss condom and contraception, may help to overcome this initial barrier of starting tough conversations. Our findings along with Chambers et al., further support the importance of incorporating parent/trusted adult-youth role play activities into sexual health programming for youth (Gavin et al., 2015; Santa Maria et al., 2015).

It is interesting to note that RCL increased youth’s reported ability to negotiate contraception use across all timepoints but not negotiate condom use across all timepoints. Specifically, 3 months (*p* = 0.212) following program completion youth in the intervention group do not report they are more likely to be able to convince their partners to use condoms than those in the control; however, at 9 months (*p* = 0.003) they are. This may be related to the large increase seen in both groups from baseline to 3 months. The increase in the control group is surprising and may be contributed to a few factors including potential contamination between the control and intervention group. Although the groups were separated throughout the camp, the community in which this program was implemented is small, and thus, it is not unlikely that youth in the intervention group interacted with youth in the control group between the end of camp and the 3 month assessment time point. The increase in ability to convince a partner to use a condom among intervention participants at 9 months may also indicate this specific item is harder to change immediately.

Furthermore, both at 3 and 9 months post intervention, youth in the intervention group did not report higher ability to refuse sex if a partner will not use a condom. While this is the only item that RCL does not significantly impact across either timepoint, it is important to explore as condom negotiation is an important contributor to actual condom use (Tschann et al., 2010; Widman et al., 2014). The ability to effectively communicate and negotiate with a partner about sexual health is paramount to condom use consistency (Stone & Ingham, 2002, Noar et al., 2002). Partner negotiation includes a level of assertiveness and skillset that might include: (1) how to bring up the condom topic, (2) when to introduce the topic, and (3) what condom negotiation strategies may be most successful (Noar et al., 2002). Not only is partner negotiation a protective factor against unwanted pregnancy and STIs, but it is also a determinant of safer sexual behavior over the lifespan (Widman et al., 2014). The RCL intervention does incorporate role play, sexual partner negotiation skills, and decision-making (Tingey et al., 2021), but these activities do not seem to influence youth’s ability to convince their partners to use a condom if the partner does not want to. The fact that the RCL intervention does not impact this item at 3 or 9 months may indicate additional work is needed to address other factors related to condom use negotiation including gender power dynamics. Our results provide rationale to further explore the delivery of components of RCL related to partner communication and/or negotiation of condom use and potentially adapt these components to improve partner negotiation skills, specifically when a partner refuses a condom.

There are several limitations to this study. First, the data collected at baseline, 3, and 9 months was self-report and may introduce response bias (Tingey et al., 2017) and response alteration based on social desirability (Mullany et al., 2013). Second, the study contained results exclusively for AI adolescents within the specific tribal nation thus limiting generalizability to other AIs and other non-AI youth. Third, because youth were young, few had initiated sex and thus the reported self-efficacy to use condoms was based on hypothetical situations for youth and not actual scenarios in which they had experienced. There is a strong limitation of not understanding behavioral outcomes (or even reported behaviors) related to partner efficacy questions. The potential threat to validity and contamination between the intervention and control group is an added limitation as the study setting is a small, close-knit community. Finally, we did not collect partner negotiation data based on relationship status which is an important component of consistent condom use (Manlove et al., 2007). Despite these limitations, this study is still a contribution to the literature and has many strengths. First, the sample specific to AI youth fills a research gap by adding to the body of AI sexual health literature.

## Conclusion

Future research should examine the long term impact of RCL on condom and contraception self-efficacy and explore how RCL impacts these items once youth initiate sex. Furthermore, additional studies should be conducted with samples of AI youth to better understand how each component of condom and contraception self-efficacy relates to actual condom and contraception use. Findings from this analysis contribute to the body of literature establishing RCL program efficacy of improving condom and contraception self-efficacy.

## Data Availability

Data is not available due to restrictions associated with tribal approval.

## References

[CR1] Akers AY, Holland CL, Bost J (2011). Interventions to improve parental communication about sex: a systematic review. Pediatrics.

[CR2] Amialchuk A, Gerhardinger L (2015). Contraceptive use and pregnancies in adolescents’ romantic relationships: role of relationship activities and parental attitudes and communication. Journal of Developmental and Behavioral Pediatrics.

[CR3] Ashcraft AM, Murray PJ (2017). Talking to parents about adolescent sexuality. Pediatrics Clinics.

[CR4] Centers for Disease Control and Prevention (2012). Diagnoses of HIV infection and AIDS in the United States and dependent areas, 2010. HIV Surveillance Report.

[CR5] Centers for Disease Control and Prevention. (2018). *Health disparities in HIV/AIDS, viral hepatitis, STDs, and TB. *Retrieved July 12, 2021, from https://www.cdc.gov/nchhstp/healthdisparities/AmericanIndians.html

[CR6] Centers for Disease Control and Prevention. (2021). *Complete listing of risk reduction evidence-based behavioral interventions*. Retrieved July 12, 2021, from http://www.cdc.gov/hiv/prevention/research/compendium/rr/complete.html

[CR7] Chambers RA, Rosenstock S, Patel H, Zhang Y, Lee A, Melgar L, Slimp A, Lee S, Susan D, Larzelere F, Tingey L (2022). Improving communication between American Indian youth and caregivers to prevent teenage pregnancy. Health Education Research.

[CR8] DiClemente RJ, Crittenden CP, Rose E, Sales JM, Wingood GM, Crosby RA, Salazar LF (2008). Psychosocial predictors of HIV-associated sexual behaviors and the efficacy of prevention interventions in adolescents at-risk for HIV infection: what works and what doesn’t work?. Psychosomatic Medicine.

[CR9] DiClemente RJ, Wingood GM, Harrington KF, Lang DL, Davies SL, Hook EW, Oh K, Crosby RA, Hertzberg VS, Gordon AB, Hardin JW, Parker S, Robillard A (2004). Efficacy of an HIV prevention intervention for African American adolescent girls: a randomized controlled trial. The Journal of the American Medical Association.

[CR10] Eaton, D. K., Kann, L., Kinchen, S., Shanklin, S., Flint, K. H., Hawkins, J., Harris, W. A., Lowry, T. M., Chyen, D., Whittle, L., Lim, C., & Wechsler, H. (2012). Youth risk behavior surveillance - United States, 2011. *Morbidity and Mortality Weekly Report: Surveillance Summaries*, *61*(4), 1–162. Retrieved July 12, 2021, from http://www.jstor.org/stable/24806047 22673000

[CR11] Fisher PA, Ball TJ (2003). Tribal participatory research: mechanisms of a collaborative model. American Journal of Community Psychology.

[CR12] Gavin, L., Moskosky, S., Carter, M., Curtis, K., Glass, E., Godfrey, E., Marcell, A., Mautone-Smith, N., Pazol, K., Tepper, N., & Zapata, L (2014). Providing quality family planning services: recommendations of CDC and the U.S. office of population affairs. *Recommendations and Reports*, *63*(4), 1–54. Retrieved July 12, 2021, from https://www.jstor.org/stable/24832591 24759690

[CR13] Gavin LE, Williams JR, Rivera MI, Lachance CR (2015). Program to strengthen parent-adolescent communication about reproductive health: a systematic review. American Journal of Preventive Medicine.

[CR14] Holtzman D, Rubinson R (1995). Parent and peer communication effects on AIDS-related behavior among U.S. high school students. Family Planning Perspectives.

[CR15] Indian Health Service (2001). Trends in Indian health 1998–1999.

[CR16] Indian Health Service (2009). Trends in Indian health, 2002–2003.

[CR17] Indian Health Service. (2014). *Trends in Indian health: 2014 edition. *Rockville (MD): IHS.

[CR18] Jaccard J, Dittus PJ, Gordon VV (2000). Parent-teen communication about premarital sex: factors associated with the extent of communication. Journal of Adolescent Research.

[CR19] Juras R, Tanner-Smith E, Kelsey M, Layzer J (2019). Adolescent pregnancy prevention: meta-analysis of federally funded program evaluations. American Journal of Public Health.

[CR20] Longmore MA, Manning WD, Giordano PC, Rudolph JL (2003). Contraceptive self-efficacy: does it influence adolescents’ contraceptive use?. Journal of Health and Social Behavior.

[CR21] Manlove J, Ryan S, Franzetta K (2007). Contraceptive use patterns across teens’ sexual relationships: the role of relationships, partners, and sexual histories. Demography.

[CR22] Manlove J, Ryan S, Franzetta K (2003). Patterns of contraceptive use within teenagers’ first sexual relationships. Perspectives on Sexual and Reproductive Health.

[CR23] Manning WD, Flanigan CM, Giordano PC, Longmore MA (2009). Relationship dynamics and consistency of condom use among adolescents. Perspectives on Sexual and Reproductive Health.

[CR24] Mullany B, Barlow A, Neault N, Billy T, Hastings R, Coho-Mescal V, Lorenzo S, Walkup JT (2013). Consistency in the reporting of sensitive behaviors by adolescent American Indian women: a comparison of interviewing methods. American Indian Alaskan Native Mental Health Research (online).

[CR25] Noar SM, Morokoff PJ, Harlow LL (2002). Condom negotiation in heterosexually active men and women: development and validation of a condom influence strategy questionnaire. Psychology & Health.

[CR26] Osterman MJK, Hamilton BE, Martin JA, Driscoll AK, Valenzuela CP, Division of Vital Statistics (2022). Births:
final data for 2020. National Vital Statistics Reports.

[CR27] Pampati S, Liddon N, Dittus PJ, Adkins SH, Steiner RJ (2019). Confidentiality matters but how do we improve implementation in adolescent sexual and reproductive Health Care?. The Journal of Adolescent Health: Official Publication of the Society for Adolescent Medicine.

[CR28] Patterson S, McDaid L, Saunders K, Battison C, Glasier A, Radley A, Stephenson JM, Johnstone A, Morelli A, Sally D, Stewart N, Cameron ST (2022). Improving effective contraception uptake through provision of bridging contraception within community pharmacies: findings from the bridge-it study process evaluation. BMJ Open.

[CR29] Raffaelli M, Bogenschneider K, Flood MF (1998). Parent-teen communication about sexual topics. Journal of Family Issues.

[CR30] Ritchwood TD, Penn D, Peaseant C, Albritton T, Corbie-Smith G (2017). Condom use self-efficacy among younger rural adolescents: the influence of parent-teen communication, and knowledge of and attitudes towards condoms. The Journal of Early Adolescence.

[CR31] Rotheram-Borus MJ, Song J, Gwadz M, Lee M, Van Rossem R, Koopman C (2003). Reductions in HIV risk among runaway youth. Prevention Science.

[CR32] Santa Maria D, Markham C, Bluethmann S, Mullen PD (2015). Parent-based adolescent sexual health interventions and effect on communication outcomes: a systematic review and meta-analysis. Perspectives on Sexual and Reproductive Health.

[CR33] St Lawrence JS, Brasfield TL, Jefferson KW, Alleyne E, O’Bannon RE, Shirley A (1995). Cognitive-behavioral intervention to reduce African American adolescents’ risk for HIV infection. Journal of Consulting and Clinical Psychology.

[CR34] StataCorp. (2021). *Stata statistical software: Release 17*. College Station, TX: StataCorp LLC.

[CR35] Stone N, Ingham R (2002). Factors affecting British teenagers’ contraceptive use at first intercourse: the importance of partner communication. Perspectives on Sexual and Reproductive Health.

[CR36] Strom Chambers RA (2021). Towards intergeneration programming: acceptability, feasibility and impact of adolescent health program including the family in indigenous communities.

[CR37] Tingey L, Chambers R, Goklish N, Larzelere F, Lee A, Suttle R, Rosenstock S, Lake K, Barlow A (2017). Rigorous evaluation of a pregnancy prevention program for American Indian youth and adolescents: study protocol for a randomized controlled trial. Trials.

[CR38] Tingey L, Chambers R, Patel H, Littlepage S, Lee S, Lee A, Davette S, Melgar L, Slimp A, Rosenstock S (2021). Prevention of sexually transmitted diseases and pregnancy prevention among Native American youths: A randomized controlled trial, 2016–2018. American Journal of Public Health.

[CR39] Tingey L, Mullany B, Chambers R, Hastings R, Lee A, Parker A, Barlow A, Rompalo A (2015). Respecting the circle of life: one-year outcomes from a randomized controlled comparison of an HIV risk reduction intervention for American Indian adolescents. AIDS Care.

[CR40] Tingey L, Sutcliffe C, Chambers R, Patel H, Lee A, Lee S, Melgar L, Slimp A, Rompalo A, Craid M, Gaydos C (2019). Protecting our future generation: study protocol for a randomized controlled trial evaluating a sexual health self-care intervention with Native American youth and young adults. BMC Public Health.

[CR41] Tomaszewski, D., Aronson, B.D., Kading, M., & Morisky, D*.* (2017). Relationship between self-efficacy and patient knowledge on adherence to oral contraceptives using the Morisky Medication Adherence Scale (MMAS-8). *Reproductive Health*, *14*, 110. 10.1186/s12978-017-0374-6 10.1186/s12978-017-0374-6 PMC5585984 28874178

[CR42] Tschann JM, Flores E, De Groat CL, Deardorff J, Wibbelsman CJ (2010). Condom negotiation strategies and actual condom use among Latino youth. Journal of Adolescent Health.

[CR43] Widman L, Noar SM, Choukas-Bradley S, Francis D (2014). Adolescent sexual health communication and condom use: a meta-analysis. Health Psychology.

